# Cleaved caspase-3 is present in the majority of glial cells in the intact rat spinal cord during postnatal life

**DOI:** 10.1007/s00418-023-02249-7

**Published:** 2023-11-08

**Authors:** R. Holota, V. Dečmanová, A. Alexovič Matiašová, J. Košuth, L. Slovinská, L. Pačut, Z. Tomori, Z. Daxnerová, J. Ševc

**Affiliations:** 1 Institute of Biology and Ecology, Faculty of Science, P. J. Šafárik University in Košice, Šrobárova 2, 04154 Košice, Slovak Republic; 2 Associated Tissue Bank, Faculty of Medicine, P. J. Šafárik University in Košice and L. Pasteur University Hospital, Tr. SNP 1, 04011 Košice, Slovak Republic; 3 Department of Regenerative Medicine and Cell Therapy, Institute of Neurobiology, Biomedical Research Center, Slovak Academy of Sciences, Šoltésovej 4, 04001 Košice, Slovak Republic; 4 Institute of Experimental Physics, Slovak Academy of Sciences, Watsonova 47, 04001 Košice, Slovak Republic

**Keywords:** Apoptosis, Cleaved caspase-3, Cleaved PARP, Spinal cord, Rat, Development

## Abstract

Cell death is an essential process that occurs during the development of the central nervous system. Despite the availability of a wide range of commercially produced antibodies against various apoptotic markers, data regarding apoptosis in intact spinal cord during postnatal development and adulthood are mostly missing. We investigated apoptosis in rat spinal cord at different stages of ontogenesis (postnatal days 8, 29, and 90). For this purpose, we applied immunofluorescent detection of two widely used apoptotic markers, cleaved caspase-3 (cC3) and cleaved poly(ADP-ribose) polymerase (cPARP). Surprisingly, we found significant discrepancy between the number of cC3^+^ cells and PARP^+^ cells, with a ratio between 500:1 and 5000:1 in rat spinal cord at all postnatal time points. The majority of cC3^+^ cells were glial cells and did not exhibit an apoptotic phenotype. In contrast with in vivo results, in vitro analysis of primary cell cultures derived from neonatal rat spinal cord and treated with the apoptotic inductor staurosporine revealed a similar onset of occurrence of both cC3 and cPARP in cells subjected to apoptosis. Gene expression analysis of spinal cord revealed elevated expression of the *Birc4 (XIAP)*, *Birc2*, and *Birc5 (Survivin)* genes, which are known potent inhibitors of apoptosis. Our data indicate that cC3 is not an exclusive marker of apoptosis, especially in glial cells, owing its possible presence in inhibited forms and/or its participation in other non-apoptotic roles. Therefore, cPARP appears to be a more appropriate marker to detect apoptosis.

**Supplementary Information:**

The online version contains supplementary material available at 10.1007/s00418-023-02249-7.

## Introduction

Caspase-3 is a member of cysteine-aspartate proteases that are considered to be important effectors of apoptosis, which represents the process of regulated cell death (Asadi et al. 2022; Taatjes et al. 2008). The very first member of this family of proteases active in cell death, termed cell death protein 3 (CED-3), was identified in *Caenorhabditis elegans*, and was reported as a homolog to murine ICE-related proteases (later known as caspases) (Yuan et al. 1993). The involvement of CED-3 and its homologs in cell death led researchers to consider that caspases are apoptotic proteins (Fraser and Evan 1997; Miura et al. 2004). During the apoptotic cascade, inactive zymogen of caspase-3 is activated by the initiator caspase-8 and/or caspase-9 (Taatjes et al. 2008). Subsequently, cleaved caspase-3 (cC3) interacts with numerous substrates in the cell, including cytoplasmic and nuclear structural proteins; molecules involved in protein synthesis and protein modification; and proteins of multiple signaling pathways controlling proliferation, differentiation, and/or cell adhesion (Fischer et al. 2003). The activity of cC3 and other effector molecules of apoptosis results in changes in cellular morphology typical for apoptotic cells, for example chromatin condensation, DNA fragmentation, formation of plasma membrane protrusions, and externalization of phosphatidylserine (Taatjes et al. 2008; Ziegler and Groscurth 2004). One of the most studied substrates of cC3 is poly(ADP-ribose) polymerase 1 (PARP-1), which is important for DNA repair and for maintenance of chromatin and genome stability (Dantzer et al. 1999). cC3 cleaves PARP-1 into two fragments, a 24-kDa fragment (p24) and 89-kDa fragment (p89), which are specific for apoptosis, unlike the other forms of cell death (Duriez and Shah 1997; Mashimo et al. 2021). Afterwards, the p24 fragment of cleaved PARP-1 (cPARP-1) enables progression of apoptosis by inhibiting DNA repair, and the p89 fragment dimerizes with and inactivates intact PARP-1 (Soldani and Scovassi 2002). Recent evidence has revealed that p89 fragment of cPARP-1 covalently binds poly(ADP-ribose) (PAR), and this complex is translocated to the cytoplasm, where it induces release and translocation of apoptosis-inducing factor (AIF) to the nucleus (Mashimo et al. 2021). Thus, p89 fragment of cPARP-1 could also be involved in AIF-mediated apoptosis.

Although cC3 is not an exclusive effector protease of apoptosis in mammals (Wilson and Kumar 2018), owing to its ability to interact with a wide spectrum of substrates (Walsh et al. 2008) it is considered the main apoptotic protease. Thus, various assays designed to identify apoptotic cells in tissue samples or in cell culture often focus on detecting activated effector caspases, especially the cC3 (Fox and Aubert 2008; Gown and Willingham 2002; Hanson and Finkelstein 2019; Takano et al. 2014). On the other hand, the presence of cC3 has been reported in various cells without typical apoptotic hallmarks (Krajewska et al. 1997). Caspase-3 activity has been linked to several non-apoptotic processes. It has been reported that cC3 is required for the differentiation of various cell types, including bone marrow stromal stem cells, osteoclasts (Szymczyk et al. 2006), skeletal muscle fibers (Fernando et al. 2002), and embryonic cells (Fujita et al. 2008), as well as to regulate the proliferation of B lymphocytes (Woo et al. 2003).

In the nervous system, apoptosis has been extensively studied because of its crucial role in embryonic development, tissue formation, the function of neuronal circuits (Yuan and Yankner 2000), and its involvement in various pathological events linked to neurodegenerative disorders (Erekat 2022) and tissue injury (Springer 2002). Despite evidence that cC3 is involved in non-apoptotic events, it is commonly used as an apoptotic marker in both older and recent studies of the spinal cord, especially under pathological conditions (Dai et al. 2019; Gülmez et al. 2022; Mirzaie et al. 2022; Nesic et al. 2001; Shen et al. 2022). On the other hand, despite the availability of wide range of commercially produced antibodies against various apoptotic markers (including cC3, cPARP, endonuclease G [EndoG], fragmented actin [fractin], cleaved caspase-7 (cC7), etc.), data regarding apoptosis in intact spinal cord during postnatal development or in adulthood are still missing. Thus, in the present study we analyzed cC3^+^ and cPARP^+^ cell populations in intact spinal cord at selected stages of ontogenesis to determine the validity of cC3 as an apoptotic marker in intact nervous tissue. We chose cC3 and cPARP from wide array of apoptotic markers as promising targets to detect apoptosis. Second, we compared the abundance of both cC3^+^ and cPARP^+^ cell populations in the spinal cords of neonatal, preadolescent, and adult rats. Third, employing an in vitro model with synchronously induced apoptosis by staurosporine treatment, we deciphered the pattern of cC3 and cPARP presence in cells undergoing apoptosis in the spinal cord tissue samples. Finally, employing gene expression analysis, we identified potential regulatory mechanisms by which cells may control cC3 activity under normal conditions in developing and mature nervous tissue.

## Material and methods

### Experimental animals

All experiments on rats were performed in accordance with the ARRIVE guidelines, the European Community Council (Directive 2010/63/EU), and current national legislation, conducted with approval from the National Food Administration of the Slovak Republic under no. Ro-3051-5/2021-220 and the Animal Care Committee of the Pavol Jozef Šafárik University in Košice. Wistar albino rats (strain Crl:WI, RRID:RGD_2308816) were obtained from Velaz (Prague, Czech Republic). The rats were housed under standard laboratory conditions with a 12-h photoperiod. The rats were fed a complete and balanced standard laboratory diet (Altromin International, Lage, Germany) and had ad libitum access to food and water. Experiments were performed on rats at postnatal day 8 (P8, neonatal period, *n* = 11), P29 (preadolescent period, *n* = 6), and P90 (young adult period, *n* = 6).

### Tissue isolation

At each age, experimental animals were terminally anesthetized with an intraperitoneal overdose of sodium thiopental (500 mg/kg). Three rats per time point and experimental group were either (i) transcardially perfused with heparinized saline for gene expression analyses or (ii) transcardially perfused with heparinized saline followed by freshly prepared 4% paraformaldehyde in 0.1 M phosphate buffer (PB) at pH 7.4 for immunofluorescent labeling.

For immunofluorescence analyses, isolated lumbar segments (L3–L5) of spinal cord were postfixed in the same fixative at 4 °C for 24 h, followed by cryoprotection in 30% sucrose. Spinal cord samples were stored at 4 °C until further processing. Isolated tissue samples were cut to 40-µm-thick coronal sections using a freezing microtome (Leica CM1850, Leica Microsystems, Mannheim, Germany). For gene expression analyses, isolated segments of lumbar spinal cord (20–50 mg) were immediately immersed in TRI Reagent™ Solution (Invitrogen, Vilnius, Lithuania, #AM9738) and stored at − 80 °C until further analysis.

### Primary cell culture preparation

Primary cell cultures were derived from spinal cords of P8 rats (*n* = 5). The rats were terminally anesthetized, decapitated, and the spinal cords were dissected. After removal of the meninges, the spinal cords were cut into small pieces and transferred to Papain Dissociation System solution (Worthington Biochemical Corporation, Lakewood, NJ, #LK003150) containing 0.01% papain and 0.01% DNase, in line with the isolation protocol, at 37 °C with intermittent gentle shaking for 45 min. After digestion, excisions were mechanically dissociated into cell suspension and centrifuged (300×*g*, 21 ℃, 10 min). The cell pellet was resuspended in DNase diluted in albumin inhibitor solution, and discontinuous density gradient (70×*g*, 21 ℃, 10 min) was used to remove membrane fragments. Subsequently, cells were seeded into 24-well tissue culture plates with laminin-coated cover glasses on the bottom (TPP, Trasadingen, Switzerland) and cultivated in culture medium composed of Dulbecco’s Modified Eagle Medium (#L0102-500) and Ham’s F12 (#L0135-500; 1/1 v/v) (both Biosera, Nuaille, France) supplemented with 5% fetal bovine serum (Biowest, Nuaillé, France, #S1400), antibiotics (penicillin/streptomycin 10,000 U/10,000 mg/mL, Biochrom AG, Berlin, Germany, #2213B), 1% B-27 supplement (#17504044), and 0.5% N-2 supplement (#17502048) (both from Gibco, Invitrogen, Carlsbad,CA, USA). Cells were cultivated under standard conditions in an incubator at 37 ℃, 5% CO_2_, and 95% humidity. In all wells, half of the medium was replaced every third day.

### Establishment of an in vitro model of an apoptotic neural cell population

The apoptotic effect of staurosporine on primary cell cultures (7 × 10^4^ cells/well) was analyzed on day 13 of the cultures. For this endeavor, 1 mM staurosporine (Cell Signaling Technology, Leiden, the Netherlands, #9953) diluted in dimethyl sulfoxide (DMSO) was added to culture medium (1:1000 dilution ratio, final concentration 1 μM dm^−3^) along with Incucyte^®^ Caspase-3/7 Dye for Apoptosis (Sartorius AG, Göttingen, Germany, #4440) and Incucyte^®^ Annexin V Dye for Apoptosis (Sartorius AG, #4641) diluted 1:1500 and 1:200, respectively.

The progression of the development and distribution of apoptotic markers in the staurosporine-induced apoptotic cell population was monitored using the IncuCyte™ ZOOM system (Essen BioScience, Ann Arbor, MI, USA) for Caspase-3/7 Dye (excitation [Ex]/emission [Em] 500/530 nm) and for Annexin V Dye (Ex/Em 593/614 nm). Analysis was performed using the IncuCyte ZOOM 2016B analysis software (Essen BioScience, RRID:SCR_019874) on nine microphotographs for each well (*n* = 4 wells) taken with a ×10 objective lens (Supplemental Table 1) every 30 min for a total of 24 h. For both markers, individual analysis was performed uniformly for each captured microphotograph (Supplemental Table 2). The results were compared with control data obtained in a similar manner from a primary cell culture that was cultivated simultaneously in staurosporine-free medium.

### Collection of samples for in vitro time-dependent analysis of apoptotic markers

On day 14 of cultivation, time-dependent analysis of the presence of apoptotic markers was conducted using primary cell culture treated with staurosporine diluted in culture medium (final concentration 1 µM dm^−3^). After staurosporine was added, primary cell cultures were fixed each hour during the cultivation, starting at 0 h (no staurosporine effect) until 7 h. Cells were fixed with freshly prepared 4% paraformaldehyde in 0.1 M PB at room temperature for 20 min. Fixed primary cell cultures were then washed with 0.1 M phosphate-buffered saline (PBS) and immunofluorescently labeled. For selection of reliable marker(s) of apoptotic cells, both intact primary cell culture (negative control) and primary cell culture 5 h after staurosporine treatment (positive control) were used for immunolabeling.

### Immunofluorescent labeling

Coronal sections of lumbar spinal cord and fixed primary cell cultures derived from spinal cord (henceforth referred to as samples) were rinsed in 0.1 M PBS. If necessary, antigen retrieval was applied by incubating the samples in 10 mM citrate buffer (pH 6.0) at 95.5 ± 0.5 °C for 10 min. After the samples were cooled to room temperature, they were washed with 0.1 M PBS. Non-specific protein activity was blocked by incubating samples with 5% normal donkey serum (NDS, Jackson Immunoresearch, West Grove, PA, USA, #017-000-12) in 0.1 M PBS with 0.3% Triton-X 100 at 4 °C overnight. Subsequently, the samples were incubated in a mixture of primary antibodies (Table 1) diluted in 0.1 M PBS containing 1% NDS and 0.3% Triton-X 100 at 4 °C overnight. After this incubation, the samples were washed with 0.1 M PBS and incubated with corresponding secondary antibodies (Table 1) diluted in 0.1 M PBS containing 1% NDS and 0.3% Triton-X 100 at room temperature in the dark for 2 h. Next, the samples were washed with 0.1 M PBS. To visualize cell nuclei, far-red dye DRAQ5 (1:500, Cell Signaling Technology, #4084) diluted in 0.1 M PBS was applied to tissue samples for 20 min. Finally, the samples were washed in 0.1 M PBS, mounted on glass slides, dried, and cover-slipped using ProLong Gold with DAPI (Invitrogen, #P36930).

**Table 1:** List of antibodies used in the study

Name	Host	Clonality	Manufacturer	Catalogue number	Lot. number	RRID	Dilution
Anti-cleaved caspase-3 (Asp175)	Rabbit	Polyclonal	Cell Signaling Technology	9661S	GR309480-1	AB_2341188	1:500
Anti-cleaved caspase-7 (Asp198) (D6H1)	Rabbit	Monoclonal	Cell Signaling Technology	8438S	Lot 3	AB_1117837	1:100
Anti-cleaved PARP (Asp214) (D6X6X)	Rabbit	Monoclonal	Cell Signaling Technology	94885	Lot 1	AB_2800237	1:100
Anti-AIF (D39D2)	Rabbit	Monoclonal	Cell Signaling Technology	5318	Lot 3	AB_10634755	1:250
Anti-EndoG	Rabbit	Polyclonal	Abcam	ab9647	GR3181009-13	AB_2098770	1:250
Anti-fractin	Rabbit	Polyclonal	Merck Millipore	AB3150	2976523	AB_262159	1:1000
Anti-actin (clone C4)	Mouse	Monoclonal	Merck Millipore	MAB1501	2951837	AB_2223041	1:200
Anti-Olig2 (clone 211F1.1)	Mouse	Monoclonal	Merck Millipore	MABN50	3128845	AB_1080741	1:200
Anti-NeuN (clone A60)	Mouse	Monoclonal	Merck Millipore	MAB377	2279235	AB_2298772	1:200
Anti-S100β	Mouse	Monoclonal	Proteintech group	66616-1-Ig	10004814	AB_2881976	1:100
Anti-APC (Ab-7) (Clone CC-1)	Mouse	Monoclonal	Calbiochem	OP80	D00150228	AB_2057371	1:200
Anti-tubulin β 3 (TUBB3) (clone TUJ1)	Mouse	Monoclonal	Covance Biolegend	801202	B233555	AB_1006340	1:500
Anti-rabbit IgG H&L (AlexaFluor^®^ 488)	Donkey	Polyclonal	Abcam	ab150073	GR3248726-1	AB_2636877	1:500
Anti-rabbit IgG H&L (AlexaFluor^®^ 555)	Donkey	Polyclonal	Abcam	ab150074	GR3241278-8	AB_2636997	1:500
Anti-mouse IgG H&L (AlexaFluor^®^ 555)	Donkey	Polyclonal	Abcam	ab150110	GR3446637-1	AB_2783637	1:500
Anti-mouse IgG H&L (AlexaFluor^®^ 594)	Donkey	Polyclonal	Abcam	ab150108	GR3360080-5	AB_2732073	1:500

### Microscopic analysis and microphotographs preparation

A Leica TCS SP5X confocal system equipped with LAS AF software (Leica Microsystems) and Leica Thunder Imager DMi8 epifluorescent microscope equipped with LAS X software (Leica Microsystems, RRID:SCR_013673) using ×10, ×40, and ×100 objective lens (Supplemental Table 1) were used to analyze immunofluorescently labeled tissue samples. Confocal imagining was done in XYZ mode (resolution 8 bits, 1024 × 1024 pixels, scanning speed 100 Hz, gain 600–750 V). The following Ex and Em wavelengths were used to visualize the fluorophores: AlexaFluor 488 (Ex 488 nm, Em 500–540 nm), AlexaFluor 555 (Ex 555 nm, Em 565–590 nm), and DRAQ5 (Ex 643 nm, Em 655–690 nm). Epifluorescence imaging was done in XYZ mode with a Leica DFC9000 GTC camera (resolution 24 bits, 2048 × 2048 pixels). The following Ex and Em wavelengths were used to visualize the fluorophores: DAPI (LED_405 filter cube Ex 405/60 nm, Em 470/40 nm) and AlexaFluor 488 (GFP filter cube, Ex 470/40 nm, Em 525/50 nm).

To analyze the cC3^+^population, five optical sections (Z-stacks) per animal at P8, P29, and P90 (*n* = 3 rats per time point) were captured using the ×40 objective lens. Stereological quantification of cC3^+^ cells was performed in ventral, lateral, and dorsal funiculi; the dorsal and ventral horns; and the central gray matter (henceforth referred to as regions of interest [ROI]) using ConfoCounter software (Institute of Experimental Physics, SAS, Košice, Slovakia, available for free download from the Microsoft Store at https://apps.microsoft.com/store/detail/confocounter/9PNSHZPXKHMM?hl=sk-sk&gl=sk&rtc=1). Densitometry analysis of the positivity of cC3^+^ nuclei was evaluated as relative intensity of fluorescence using Ellipse 2.0 software (ViDiTo, Košice, Slovakia). The abundance of cPARP^+^ cells in the spinal cord was counted using a ×40 objective lens on ROI of 20 tissue slices per P8, P29, and P90 spinal cords (*n* = 3 animals per time point).

The IncuCyte™ ZOOM system (Essen BioScience) with ×10 and ×20 objective lens was used to analyze primary cell cultures (Supplemental Table 1). The following parameters for imaging were used: resolution 24 bits, 1392 × 1040 pixels, Ex 460/40 nm and Em 524/40 nm, and Ex 585/40 nm and Em 665/80 nm filter.

Microphotographs were assembled into figures using ImageJ 1.53t (NIH, Bethesda, MD, USA, RRID:SCR_003070), Adobe Illustrator (RRID:SCR_010279), and Adobe Photoshop (RRID:SCR_014199) (Adobe Systems, San Jose, CA, USA). All modifications were limited to cropping and adjustment of brightness and contrast.

### Identification of cC3 and cPARP after staurosporine-induced apoptosis in the in vitro model

Immunofluorescent co-localization using anti-tubulin β 3 (clone TUJ1) and anti-APC (clone CC1) antibodies was realized to analyze the presence of cC3 and cPARP in neuronal and oligodendroglial populations of primary cell culture treated with staurosporine. Uniform analysis procedure (Supplemental Table 2) was prepared with the IncuCyte ZOOM 2016B analysis software (Essen BioScience) and used to estimate the number of cC3^+^ and cPARP^+^ cells per mm^2^ in 14 microphotographs taken with a ×10 objective lens from each well (*n* = 6 wells per experimental group). Co-localization of apoptotic (cC3, cPARP) and phenotypic (TUJ1, CC1) markers in cells was identified as signal overlap and performed on three wells per experimental group. Co-localization was evaluated as percentage of neurons or oligodendroglial cells.

### Analysis of gene expression

Gene expression was analyzed to evaluate the abundance of inhibitors of apoptosis (IAPs, encoded by *Birc* genes) in the spinal cord of P8, P29, and P90 rats (*n* = 3 rats per time point). The absolute number of each gene transcript was determined by digital PCR (dPCR). The analyzed cDNA was prepared by reverse transcription (RT) of total RNA, isolated using the TRI Reagent™ Solution. Anchored oligo dT primer and RevertAid Reverse Trancriptase (Thermo Scientific, Vilnius, Lithuania, #EP0441) was used for RT. Both protocols were performed according to the manufacturer’s instructions. dPCR was performed by using the nanoplate-based technology QIAcuity Digital system, the QIAcuity One 5-plex instrument, 8.5K QIAcuity™ Nanoplates (#250021), and QIAcuity™ EG PCR Kit (#2500112) (all from Qiagen, Hilden, Germany) for amplification and detection of the products. Gene-specific PCR primers (Supplemental Table 3) were designed according to the reference sequence of rat *Birc1-Birc7* genes by Primer-Blast (RRID:SCR_003095). Amplification of the desired product and lack of primer-dimers was confirmed by agarose gel electrophoresis and quantitative reverse transcription PCR (RT-qPCR). Depending on the gene, 10–50 ng of RNA/cDNA per 12-µL reaction was amplified by two-step PCR: denaturation at 95 °C for 2 min followed by 35 cycles of 95 °C for 15 s, and 60 °C for 15 s. To verify that the detection/quantification occurred in the linear range, a portion of each sample was diluted twofold and analyzed alongside the undiluted sample. RNA/cDNA isolated from testes of P29 rats was used as a positive control for transcripts with a low amount or absent PCR product in the spinal cord samples. Finally, the detected numbers of the individual *Birc* gene transcripts were normalized to normalization factor (NF) based on the expression of two reference genes (*Gapdh* and *Eef1a1*), which are stably expressed during postnatal development (Košuth et al. 2020). The NF was calculated as the geometric mean of copies of both reference genes. For genes with very low or absent transcripts in the spinal cord, the limit of quantification (LOQ) was determined. Serial dilutions of RNA/cDNA isolated from rat testes (which has high expression of *Birc* genes) were used for the purpose. The LOQ of *Birc1*, *Birc3*, and *Birc7* transcripts per NF were 3.1–3.7 × 10^−5^, 2.2–2.6 × 10^−4^, and 1.5–1.6 × 10^−4^, respectively.

### Statistics

GraphPad Prism (version 9.0.0, GraphPad Software, San Diego, CA, USA, RRID:SCR_002798) was used for statistical analysis and to prepare graphs. The data were analyzed using unpaired *t* test, one-way analysis of variance (ANOVA) followed by the Tukey–Kramer post hoc test for multiple comparisons or repeated-measures two-way ANOVA followed by the Bonferroni post hoc test for multiple comparisons. A difference between groups or time points was considered statistically significant with a *p* value of less than 0.05.

## Results

### Selection of appropriate apoptotic marker(s)

**Fig. 1:**
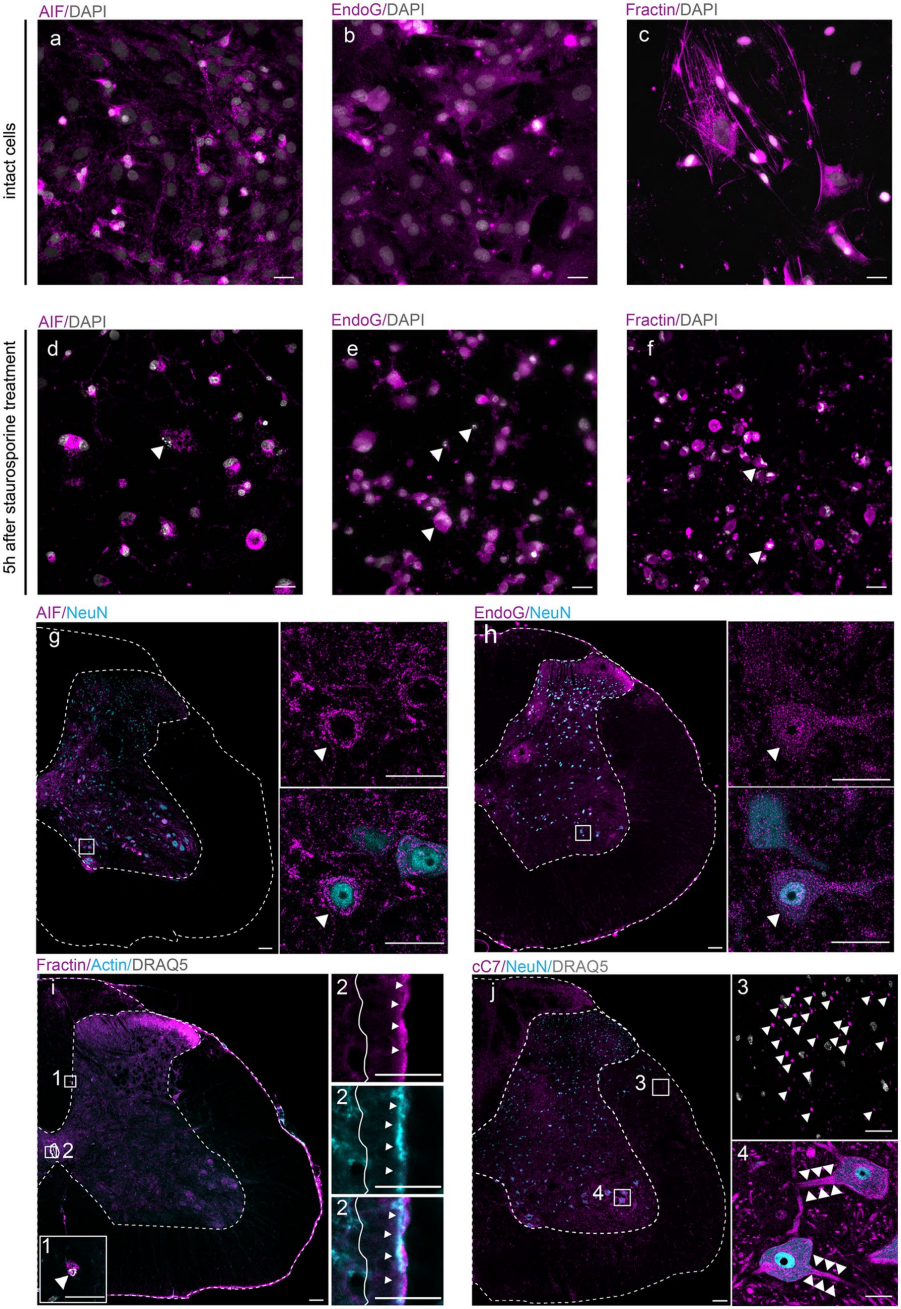
Detection of apoptotic markers in untreated and staurosporine-treated primary cell cultures and in intact spinal cord. Microphotographs of intact primary cell cultures (**a**–**c**; negative control) and primary cell cultures 5 h after staurosporine treatment (**d**–**f**; positive control) immunolabeled with anti-AIF (**a**, **d**), anti-EndoG (**b**, **e**), and anti-fractin (**c**, **f**) antibodies (magenta) with highlighted nuclei (DAPI, gray). Apoptotic morphology is indicated by arrowheads. Microphotographs of P90 rat spinal cord labeled with anti-AIF (**g**) and anti-EndoG (**h**) antibodies (magenta) and anti-NeuN antibody (cyan). White squares indicate motor neurons. Microphotographs of P90 rat spinal cord labeled with anti-fractin (magenta) and anti-actin (cyan) antibodies (**i**), with detailed pictures of fractin^+^ cell with a fragmented nucleus (DRAQ5, gray) (**i1**) and apical cytoplasm of ependymal cells positive for fractin and actin (**i2**). Microphotographs of P90 rat spinal cord labeled with anti-cC7 antibody (magenta) and anti-NeuN antibody (cyan). cC7 identified in axons (white arrowheads) of white matter in the lateral funiculus (**j3**) and in the cytoplasm and processes of motor neurons of ventral horn (**j4**). The scale bar is 25 µm in **a**–**f** and **g**–**j** (insets) and 100 µm in **g**–**j** (main)

**Fig. 2:**
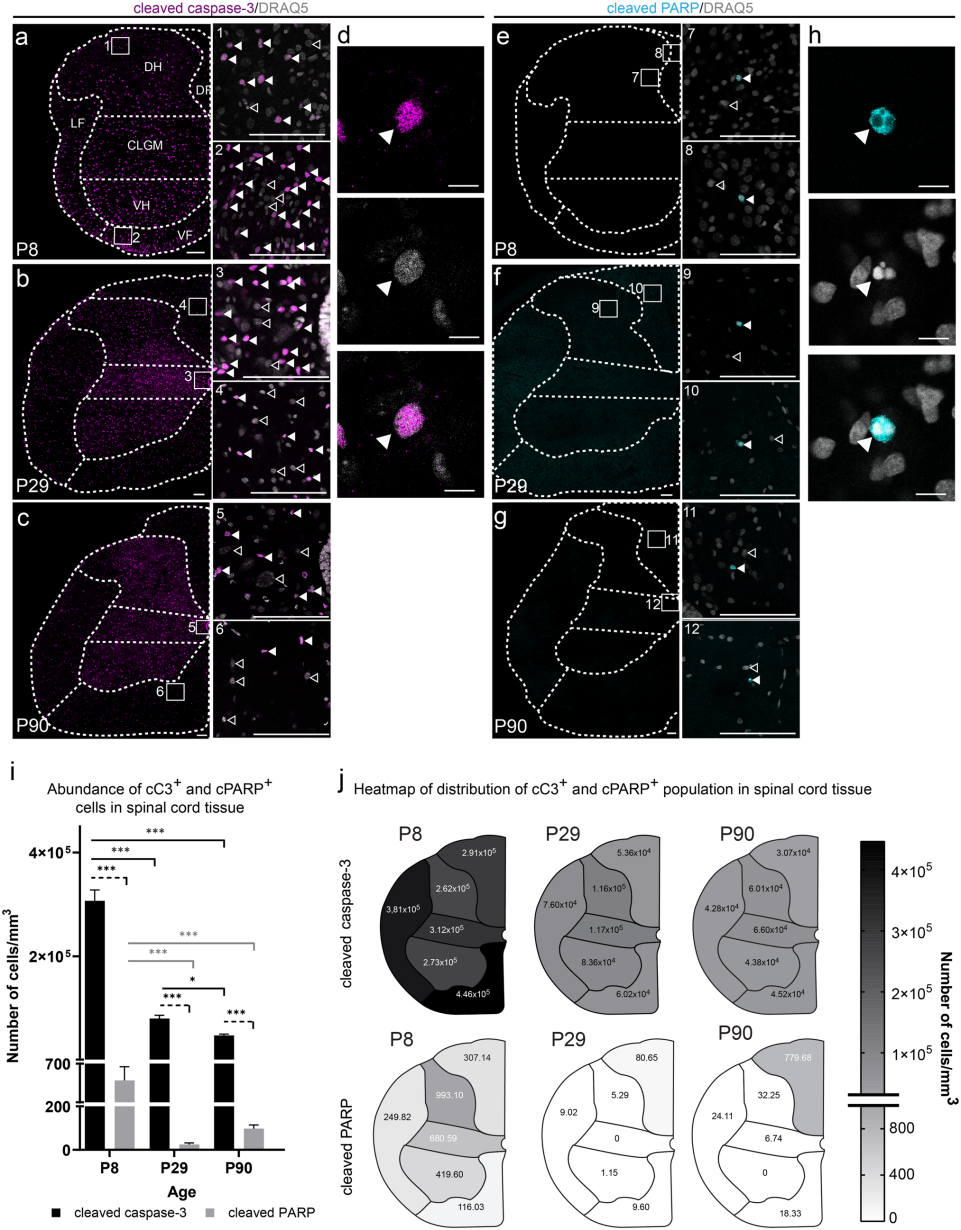
Analysis of aC3^+^ and cPARP^+^ populations in postnatal rat spinal cord. Representative microphotographs of a single optical section of spinal cords from P8 (**a**), P29 (**b**), and P90 (**c**) rats with detailed microphotographs of selected ROI showing the abundance and distribution of cC3^+^ cells (magenta) (1–6). The detailed microphotograph shows the typical morphology of cC3^+^ (magenta) and DRAQ5^+^ nucleus (gray) (**d**). Representative microphotographs of a single optical section of spinal cords from P8 (**e**), P29 (**f**), and P90 (**g**) rats with detailed microphotographs of selected ROI showing the abundance and distribution of cPARP^+^ cells (cyan) (7–12). The detailed microphotograph shows fragmented cPARP^+^ (cyan) and DRAQ5^+^ nucleus (gray) (**h**). Comparison of the populations studied in the P8, P29, and P90 rat spinal cords (**i**). The overall number of cells in both groups is expressed as a weighted average ± standard deviation of the individual ROI. Statistical comparisons were made between the cC3^+^ and cPARP^+^ populations within the analyzed ages (dashed black line; unpaired *t* test). The changes in the cC3^+^ cells (black line) and cPARP^+^ cells (gray line) during ontogenetic development were analyzed using one-way ANOVA. Heatmaps of distribution of cC3^+^ and cPARP^+^ cells in analyzed ROI in spinal cord with corresponding mean value of cells/mm^3^ (**j**). *VF* ventral funiculus, *LF* lateral funiculus, *DF* dorsal funiculus, *DH* dorsal horn, *CLGM* central and lateral gray matter, *VH* ventral horn. Statistical significance: *ns* not significant; *p ≤ 0.05; **p ≤ 0.01; ***p ≤ 0.001. The scale bar is 100 µm in **a**, **b**, **c**, **e**, **f**, **g** and 10 µm in** d** and** h**

To explore the populations of cells undergoing apoptosis in intact spinal cord during postnatal development and adulthood, for the first step we decided to select appropriate marker(s) that should be clearly targeted with commercially available antibodies. With respect to the requirement of unambiguous interpretation of immunofluorescence labeling, the optimal apoptotic markers should meet two basic criteria: specificity for apoptotic cells and the discrete appearance of the signal restricted optimally to the nucleus. We tested widely used antibodies against AIF, endonuclease G (EndoG), fragmented actin (fractin), cC3, cleaved caspase 7 (cC7), and cPARP (Table 1) in the following samples: (i) intact primary in vitro culture derived from spinal cord (negative control), (ii) primary in vitro culture treated with the inductor of apoptosis - staurosporine (positive control), and (iii) coronal sections of intact adult rat spinal cord (Fig. 1a–j). AIF and EndoG are involved in caspase-independent apoptotic pathways. The anti-AIF antibody detects endogenous levels of total AIF protein present in the sample. The anti-EndoG antibody recognizes mitochondrial EndoG protein, which should be detectable in the nucleus during apoptosis (manufacturerʼs datasheet information; Li et al. 2001; Joza et al. 2009). Both AIF and EndoG were localized in cytoplasm of cells in the primary cell culture with or without apoptotic morphology (i.e., pyknotic, semilunar, and shattered nucleus) (Fig. 1a, b, d, e). However, we also detected AIF and EndoG in the cytoplasm of cells in the spinal cord sections, most notably in motor neurons (Fig. 1g, h). Similarly, we detected signal for the anti-fractin antibody, which detects fragmented form of actin caused by caspase activity during apoptosis, in both cells with normal morphology (Fig. 1c) and cells with apoptotic morphology (Fig. 1f) in primary cell culture. We also detected co-localization of anti-fractin and anti-actin signals in the ependymal cells of the central canal in the spinal cord samples, indicating possible nonspecific binding of the anti-fractin antibody to both fragmented (Fig. 1i1) and normal (Fig. 1i2) forms of actin. We noted cC7 signal in axons of white matter and cytoplasm of motor neurons (Fig. 1j). In contrast to the apoptotic markers mentioned above, cC3 signal was located predominantly in cell nuclei, similarly as in other studies (Oomman et al. 2006; Keistler et al 2017). The anti-cC3 antibody labeled mostly nuclei in cell culture and only nuclei in tissue samples with variable intensity, with or without apoptotic morphology (Fig. 2a–d). On the basis of the results of our densitometric analysis, we classified the nuclei into two categories: “low-intensity signal” and “high-intensity signal” with relative fluorescent intensity of 9.23% ± 1.39% and 26.3% ± 6.92%, respectively (Supplemental Fig. 1a, b). For the purpose of the study, we only considered nuclei with high-intensity signal as positive. We observed immunofluorescent signal for the antibody against p89 fragment of PARP (cPARP) mostly as a perinuclear ring in cells with apoptotic hallmarks, with no or only low background signal (Fig. 2e–h). Taken together, our analysis revealed that cPARP could represent a proper marker of apoptosis in the spinal cord, giving strong signal in fragmented cells, while cC3 seems to be present in broad populations of cells with morphology ranging from normal to fragmented. Variation in the morphology of cC3^+^ cells and discrepancy in their quantity compared to cPARP^+^ cells could be explained by early caspase-3 activation and late PARP cleavage during apoptosis. Therefore, in the subsequent analyses we focused on characterization of aC3^+^ and cPARP^+^ cell populations.

### cC3^+^ cells are much more abundant in the spinal cord than cPARP^+^ cells

In the next step, we focused on detecting apoptotic cells in intact spinal cord at several stages of ontogenesis: the neonatal period (P8), the preadolescent period (P29), and adulthood (P90). According to our immunofluorescence analysis, there is large discrepancy in the number of cC3^+^ and cPARP^+^ cells and changes in population abundance during rat ontogenesis (Fig. 2a–j). On the basis of the results of quantification analysis, we calculated that the ratio of cC3^+^ to cPARP^+^ cells in the spinal cord is 593:1 (P8), 4490:1 (P29), and 487:1 (P90) (Fig. 2i). The cC3^+^ cell population was the most prevalent in P8 rats (307,212.83 ± 20,710.03 cells/mm^3^) and diminished significantly in the later phases of ontogenesis (P29, 80,166.35 ± 6428.40 cells/mm^3^; P90, 47,518.84 ± 20,7410.03 cells/mm^3^). cPARP^+^ cells were also most prevalent in P8 spinal cord (613.40 ± 70.936 cells/mm^3^), relative to P29 (24.33 ± 7.37 cells/mm^3^) and P90 (97.51 ± 16.85 cells/mm^3^).

In addition to differences in the abundance of cC3^+^ and cPARP^+^ cells, we observed differences in the distributions of both populations considering the ROI of the spinal cord coronal sections (Fig. 2j). At P8, the majority of cC3^+^ cells were in the white matter (mainly in the ventral and lateral funiculi). At P29 and P90, cC3^+^ cells were most abundant in the gray matter, mainly in the dorsal horn and the central and lateral gray matter. On the contrary, cPARP^+^ cells were most abundant in the dorsal horn at P8. The abundance of these cells decreased in all ROI during the later stages of ontogenesis, with the exception of the dorsal funiculus of P90 rats.

The significant differences in the abundance and distribution of cC3^+^ and cPARP^+^ cells confirm the presumption that unlike cPARP, cC3 should appear earlier and possibly for a longer time in a cell progressing through the apoptotic cascade. Therefore, the cC3^+^ population could be the more abundant group of cells with early caspase-3 activation relative to a significantly smaller population of cells with late cleavage of PARP during apoptosis. The other explanation for significantly larger population of cC3^+^ cells could be either the inhibition of cC3 or possible non-apoptotic functions of cC3 in spinal cord cells. To explore this potentiality, in the next step we examined the presence of both cC3 and cPARP in cells after apoptotic stimulus in an in vitro primary cell culture established from P8 rat spinal cord.

### Use of an in vitro apoptotic model to determine the involvement of cC3 and cPARP throughout the progression of the apoptotic cascade

**Fig. 3:**
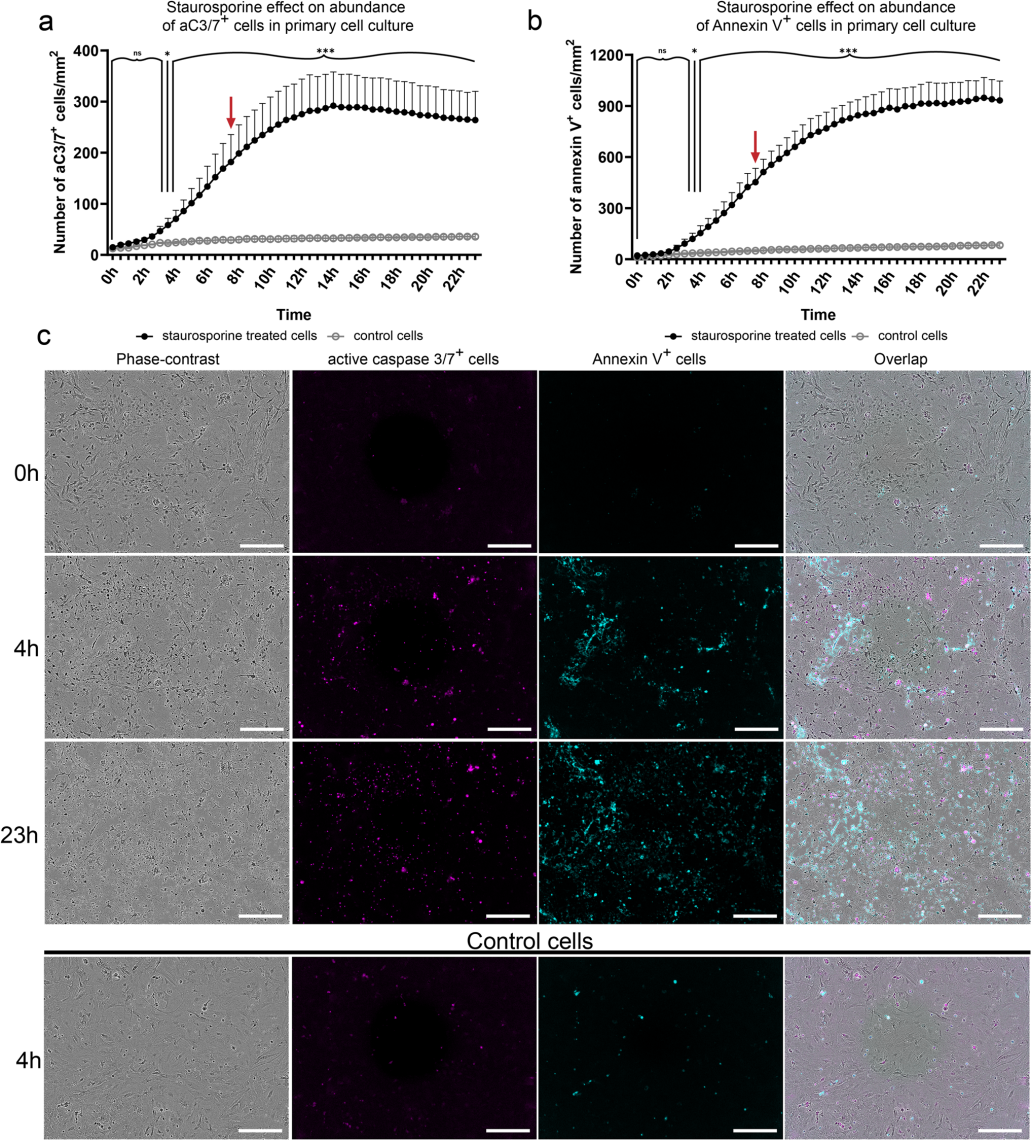
Analysis of the staurosporine effect on primary cell culture derived from P8 spinal cord. The effect of staurosporine treatment on primary cell cultures over a 24-h period was measured by activation of caspase-3/7 (**a**) and externalization of phosphatidylserine (Annexin V) (**b**). The interval of Annexin V^+^ cells overgrowth relative to aC3/7^+^ population is indicated by red arrow (**a**, **b**). Primary cell culture without staurosporine was used as a negative control. Abundance is expressed as mean ± standard deviation (cells/mm^2^). Changes in abundance of cells were compared to 0 h of staurosporine treatment (unaffected by staurosporine; repeated measures two-way ANOVA). Representative microphotograph of primary cell cultures in selected time points (**c**). Microphotographs show phase contrast, aC3/7^+^ cells (magenta) and Annexin V^+^ cells (cyan). Statistical significance: *p ≤ 0.05; **p ≤ 0.01; ***p ≤ 0.001. The scale bar is 300 µm

Given the significant differences in the number of cC3^+^ and cPARP^+^ cells in postnatal rat spinal cord, we focused on determining whether this discrepancy in the population sizes reflects differences in the time windows when these markers are detectable in cells undergoing apoptosis. To identify the period when cC3 and cPARP are detectable in apoptotic cells, we performed an in vitro evaluation on caspase-3 activation and PARP cleavage using primary cell culture isolated from P8 spinal cord. To obtain synchronous populations of apoptotic cells, we treated the cells with staurosporine, which should trigger apoptosis. We took advantage of real-time live-cell imaging and analysis using the IncuCyte™ ZOOM system to get an initial overview regarding the effect of staurosporine on the primary cell culture. Effect of staurosporine treatment was analyzed over a 24-h period by using a Casp3/7 dye (indicating activation of both caspase-3 and caspase-7; aC3/7^+^) and an Annexin V dye (indicating the externalization of phosphatidylserine) (Fig. 3a–c). Compared with 0 h (i.e., the time just before induction of apoptosis), we found a significant increase in the number of cells with active effector caspases as well as cells with externalized phosphatidylserine 3.5 h after staurosporine treatment. The number of aC3/7^+^ cells reached a plateau at 9 h, while the number of Annexin V^+^ cells continued to rise. On the basis of a statistical comparison between the numbers of aC3/7^+^ cells and Annexin V^+^ cells, we noted significant overgrow of the Annexin V^+^ population 7.5 h after staurosporine administration (two-way ANOVA, *p* < 0.05). We presume that higher numbers of Annexin V^+^ cells in later intervals indicate the presence of alternative forms of regulated and/or unregulated cell death (Shlomovitz et al. 2019). Therefore, to reduce the impact of other forms of cell death on our measurements, we decided to investigate whether there is a time delay between caspase-3 activation and PARP cleavage in cells during the first 7 h after the administration of staurosporine. Thus, we could potentially explain the difference in the temporal distribution of cC3 and cPARP in cells undergoing apoptosis in the spinal cord.

**Fig. 4:**
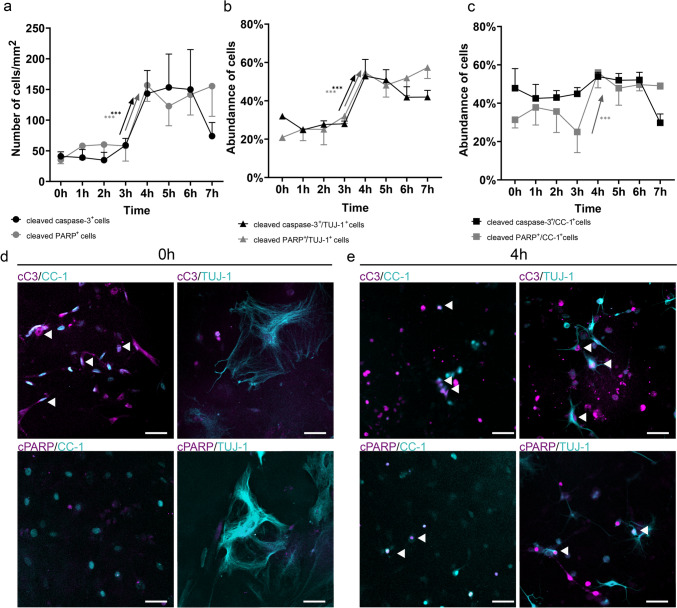
Immunofluorescence analysis of the presence of cC3 and cPARP in fixed, staurosporine-treated primary cell culture. Time-dependent analysis of the abundance of cC3^+^ and cPARP^+^ cells in staurosporine-treated primary cell culture (**a**). The abundance of both markers is expressed as mean ± standard deviation (cells/mm^2^). Compared to 0 h (no staurosporine effect, ANOVA), there was an increase in the number of cC3^+^ cells and cPARP^+^ cells at the same time point. Time-dependent analysis of changes in the proportion of cC3^+^/TUJ-1^+^ and cPARP^+^ /TUJ-1^+^ neurons (**b**) and cC3^+^/CC-1^+^ and cPARP^+^ /CC-1^+^ oligodendrocytes (**c**) in the whole TUJ-1^+^ neuronal or CC-1^+^ oligodendroglial populations (ANOVA). TUJ-1^+^ neurons showed a similar distribution of both apoptotic markers as the whole primary cell culture at the examined time points after staurosporine treatment. While cPARP^+^ /CC-1^+^ oligodendrocytes showed a similar distribution as whole primary cell culture, we observed greater representation of cC3^+^/CC-1^+^ cells from the beginning of experiment. The increased abundance is indicated with a black arrow (cC3^+^ cells) and gray arrow (cPARP^+^ cells), respectively. Representative microphotographs of primary cell cultures at 0 h (intact, **d**) and 4 h (**e**) after staurosporine administration. Apoptotic markers (cC3, cPARP) are shown in magenta, and phenotypic markers (TUJ-1, CC-1) are shown in cyan. Arrowheads show co-localization in neuronal and oligodendroglial cells. Statistical significance: *p ≤ 0.05; **p ≤ 0.01; ***p ≤ 0.001. The scale bar is 50 µm

Subsequently, we investigated the temporal relationship between caspase-3 activation and PARP cleavage in primary cell culture at 0–7 h after the induction of apoptosis with staurosporine. According to our results, the number of cC3^+^ (in line with our previous experimental data) and cPARP^+^ cells increased significantly at 4 h after induction of apoptosis (Fig. 4a). Thus, our data indicate the onset of presence of detectable levels of cC3, as well as cPARP in cells at approximately the same time: 3.5 h from the induction of apoptosis.

We then investigated whether there are differences between the induction of apoptosis and the onset of presence of cC3 and cPARP in two major cell phenotypes present in the primary cell culture that are sensitive to staurosporine: neurons (identified by the anti-tubulin β 3 [clone Tuj-1] antibody, which recognize tubulin of microtubules present in neurons) and oligodendroglial cells (identified by the anti-adenomatosis polyposis coli [clone CC-1] antibody, which recognize oligodendrocyte cell bodies; Lang et al. 2013; Ševc et al. 2014; Huang et al. 2023) (Fig. 4b, c). Our analyses revealed that TUJ-1^+^ neurons showed a similar distribution of both apoptotic markers as the whole primary cell culture in examined time points after staurosporine treatment (Fig. 4b, d, e). On the other hand, we noticed a high proportion of cC3^+^/CC-1^+^ oligodendrocytes even before the induction of apoptosis and during the subsequent period until 3 h (Fig. 4c–e). At 4 h, the number of cC3^+^/CC-1^+^ oligodendrocytes increased only moderately, in contrast to PARP^+^ oligodendrocytes, the levels of which followed a similar pattern to the whole population and TUJ-1^+^ neurons (Fig. 4a, b).

On the basis of the data obtained from the in vitro model, we assume that both temporal activation of caspase-3 and cleavage of PARP in the studied populations occur at similar time points after the induction of apoptosis. Our results indicate that because of the similar time windows, when cC3 and cPARP are present in cells undergoing apoptosis, the size of the cC3^+^ and cPARP^+^ populations should also be similar in tissues, if both markers are involved solely in the apoptotic cascade. Thus, as a result of the significant discrepancy between the size of the cC3^+^ and cPARP^+^ populations in the spinal cord, we concluded that cC3 may be inhibited or involved in other non-apoptotic processes

### Caspase-3 activation occurs mostly in glia of postnatal rat spinal cord

Because in vitro analyses revealed possible activation of caspase-3 in non-apoptotic cells, especially in glial cells, we decided to examine whether phenotypic representation of cC3^+^ population identified in the in vitro experiments resembles real conditions in the spinal cord.

**Fig. 5:**
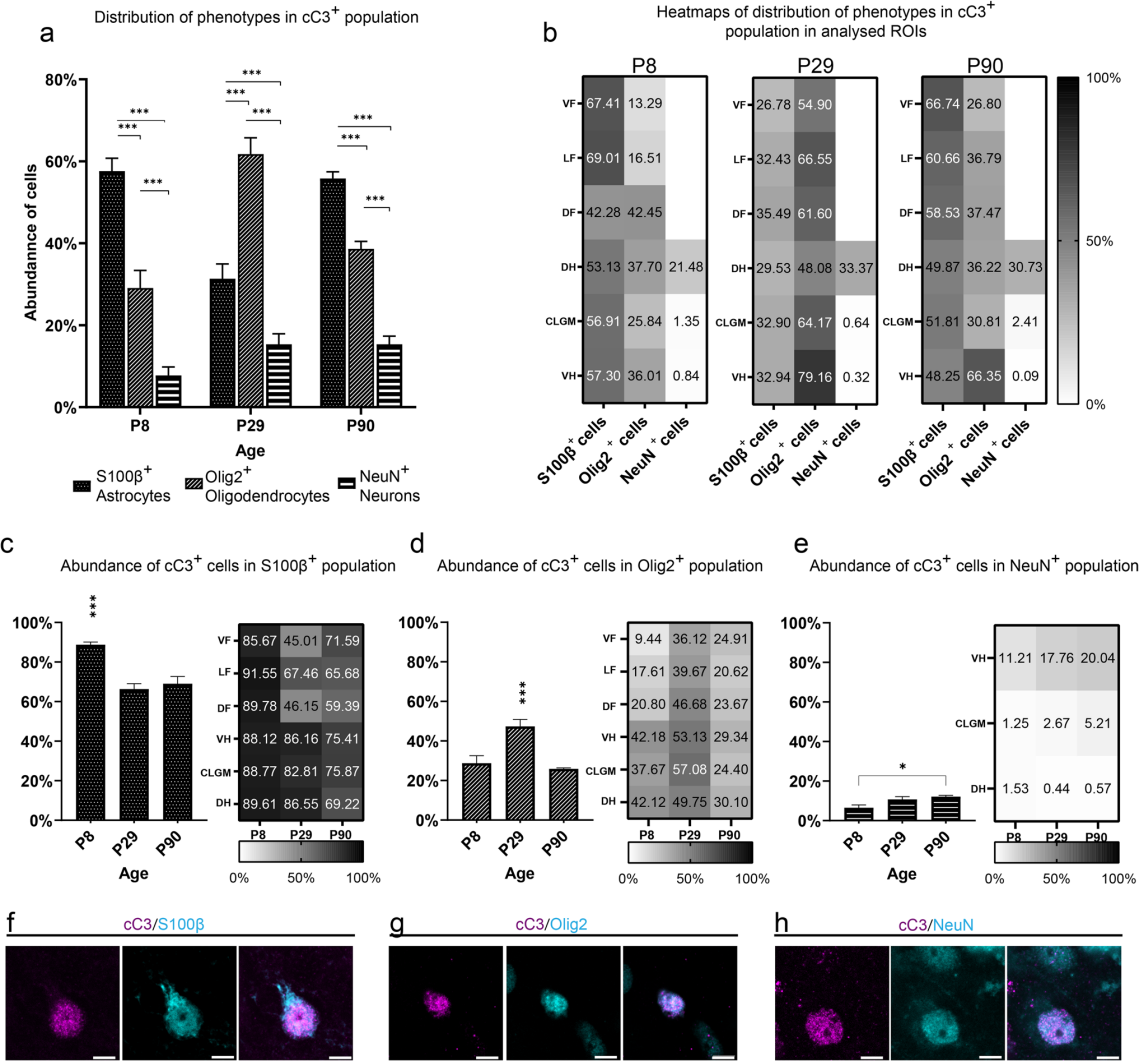
Analysis of the phenotypic composition of the cC3^+^ population. Comparison of the phenotypic representation of the cC3^+^ population in P8, P29, and P90 rat spinal cord (**a**). The phenotypic representation is expressed as the mean with standard deviation (%) of all analyzed cC3^+^ cells and was statistically compared between the ages (one-way ANOVA). Heatmaps displaying the distribution of the analyzed phenotypes of cC3^+^ population within ROI (**b**). Comparison of the abundance of cC3^+^ cells within the whole tissue and distribution of cC3^+^ cells in ROI within the populations of S100β^+^ astrocytes (**c**), Olig2^+^ oligodendrocytes (**d**), and NeuN^+^ neurons (**e**). The abundance of cC3^+^ cells is presented as the mean ± standard deviation (%) of all S100β^+^ astrocytes, Olig2^+^ oligodendrocytes, and NeuN^+^ neurons (one-way ANOVA). Representative microphotographs showing cC3^+^/S100β^+^ astrocyte (**f**) and cC3^+^/Olig2^+^ oligodendrocyte (**g**) and cC3^+^/NeuN^+^ neuron (**h**). Statistical significance: *p ≤ 0.05; **p ≤ 0.01; ***p ≤ 0.001. The scale bar is 5 µm

Co-localization analysis of cC3 and selected phenotypic markers of neurons, oligodendrocytes, and astrocytes proved that the cC3^+^ population of the spinal cord is primarily composed of glial cells (Fig. 5a–h). Surprisingly, unlike the cC3^+^ populations in the spinal cords of P8 and P90 rats, which predominantly consist of S100β^+^ astrocytes, the cC3^+^ population in P29 rats was composed mostly of Olig2^+^ oligodendrocytes (Fig. 5a). Single ROI cC3^+^ composition analysis revealed the same result as the whole tissue analysis. We noted only a few exceptions. cC3 was almost absent in the population of oligodendrocytes in the ventral and lateral funiculi of P8 rat spinal cord. On the other hand, approximately 66–79% of oligodendrocytes in the ventral horns contained cC3 at P29 and P90. Compared with glial cells, NeuN^+^ neurons represented a smaller portion of the spinal cord cC3^+^ population. At all ages studied, we identified cC3^+^/NeuN^+^ neurons mostly in the dorsal horns (Fig. 5b).

Furthermore, examining the abundance of cC3^+^ cells in individual populations provided even more intriguing results. In the S100β^+^ astroglial population, a substantial portion of all analyzed cells were cC3^+^. At P8, most S100β^+^ cells co-localized with cC3 and were highly abundant in all analyzed ROI. In the central and lateral gray matter, almost all analyzed astrocytes were cC3^+^. Even though the number of cC3^+^ astrocytes decreased significantly in the later stages of ontogenesis, they still represented most astrocytes in almost all analyzed ROI (Fig. 5c).

A significant number of cC3^+^ cells can also be seen in the Olig2^+^ oligodendroglial population. Around a quarter of all cells of the P8 and P90 rat spinal cord were cC3^+^. While the proportion of cC3^+^/Olig2^+^ oligodendrocytes was comparable within the ROI studied at P29 and P90, there was a greater proportion of cC3^+^/Olig2^+^ cells in the ROI of gray matter of P8 spinal cord (Fig. 5d). Interestingly, only 9.44% of Olig2^+^ oligodendrocytes in the P8 ventral funiculus were also cC3^+^. In the NeuN^+^ neuronal population, we identified only a small portion of cC3^+^ cells, with significantly higher representation only in the dorsal horn of gray matter (Fig. 5e). Taken together, our data indicate that caspase-3 is activated in only a small population of neurons, while most glial cells, especially astrocytes, contain detectable levels of cC3. Compared to cC3^+^ cells, the morphology in most cPARP^+^ cells of the spinal cord resembled the apoptotic phenotype with a fragmented cell body. In contrast to cPARP^+^ cells, most of the aC3^+^ cells had normal morphology, indicating that activation of caspase-3 in rat spinal cord could play an important role in other processes, especially in glia.

### Activity of cC3 in rat spinal cord may be regulated by specific IAP proteins

Given the number of cells with detectable levels of cC3^+^ present in the spinal cord on one side, and the small number of cells with the apoptotic phenotype, we presumed that there could be some mechanisms regulating caspase activity in those cC3^+^ cells, which obviously do not undergo apoptosis. One possible explanation could be the inhibition of cC3 activity by specific proteins known as inhibitors of apoptosis (IAPs), which could maintain cC3 in steady state in the cells. Therefore, we decided to examine the expression of all known *Birc* genes (*Birc1 to Birc7*), which encode the members of the IAP family, in the rat spinal cord at all selected ontogenetic stages (P8, P29, and P90). We used dPCR to determine their absolute transcript levels.

**Fig. 6:**
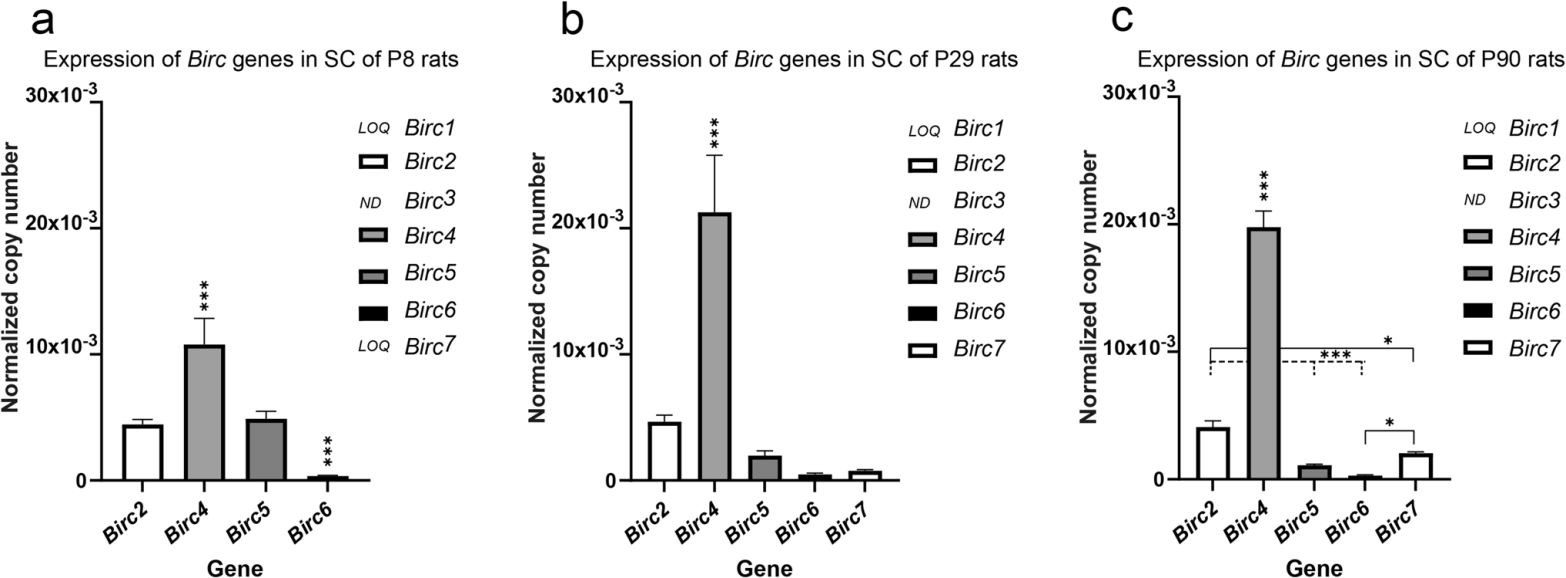
Expression of *Birc* genes encoding IAPs in the rat spinal cord. The absolute quantities of *Birc* transcripts in spinal cord at P8 (**a**), P29 (**b**), and P90 (**c**) detected by dPCR. The normalized copy number represents the number of *Birc* transcripts per normalization factor derived from the mean of expression of two reference genes—*Gapdh* and* Eef1a1*. The data are expressed as the mean ± standard deviation of three animals. *LOQ* under the limit of quantification, *ND* non-detected. Statistical comparison was performed by one-way ANOVA. Statistical significance: *p ≤ 0.05; **p ≤ 0.01; ***p ≤ 0.001

We found that the majority of the IAPs (*Birc1*, *Birc3*, *Birc6*, and *Birc7*) had very low expression in the rat spinal cord (Fig. 6a–c). On the other hand, the most abundant gene transcripts were *Birc4*, *Birc2*, and *Birc5*. Collectively, these three IAPs constituted more than 90% of the entire IAP gene family (98% at P8, 96% at P29, and 91% at P90). The most highly expressed IAP was *Birc4* (*XIAP*): its transcripts comprised more than 50% of all IAP gene transcripts at P8 and increased to more than 70% at P29 and P90. The *Birc4* transcriptional level was approximately two times (at P8) or even four times (at P29 and P90) higher than the level of *Birc2. Birc2*, another highly expressed gene, reached an expression level representing 15% at P8 and 20% of all IAP transcripts at P29 and P90. The amount of this relatively abundant gene transcript seems to be very steady in the spinal cord, without apparent change during postnatal life. Similarly high copy numbers of *Birc2* and *Birc5* (*Survivin*) were recorded, but the latter only in neonatal spinal cord. In older individuals, *Birc5* transcription gradually decreased (2.5-fold at P29 and 4.5-fold at P90). In comparison with other IAPs, *Survivin* represented the only gene with negative regulation during ontogenesis. Taken together, our analysis proved the presence of considerable levels of several IAPs (*Birc4*, *Birc2*, and *Birc5*) in the spinal cord, which could be responsible for inhibiting caspase-3 activity. This could be a possible explanation for the discrepancy between the number of cPARP^+^ and cC3^+^ cells in the rat spinal cord during postnatal life.

## Discussion

Owing to their critical role in development and pathological states of spinal cord (Erekat 2022; Springer 2002; Yuan and Yankner 2000), both apoptosis and caspase-3 have been intensively investigated topics of neuroscience. cC3, regarded as a crucial protein of apoptosis (Lakhani et al. 2006; Walsh et al. 2008), interacts with numerous proteins, leading to characteristic changes in cellular morphology and biochemistry (Taatjes et al. 2008). At the same time, multiple studies have reported caspase-3 activity in non-apoptotic processes (Fernando et al. 2002; Fujita et al. 2008; Miura et al. 2004; Szymczyk et al. 2006; Woo et al. 2003). Nevertheless, numerous studies have investigated apoptosis in the spinal cord, especially under pathological conditions, by detecting caspase-3 activation (Dai et al. 2019; Gülmez et al. 2022; Mirzaie et al. 2022; Nesic et al. 2001; Shen et al. 2022). On the other hand, studies investigating the apoptotic processes in the intact developing nervous system by employing the commercially available antibodies are lacking. In the present study, we provided a comprehensive analysis of the cC3^+^ cell populations in the rat spinal cord at selected stages of postnatal life by utilizing in vivo and in vitro models. Our finding challenged the relevance of cC3 as an apoptotic marker for non-pathological conditions.

To revise the relevance of cC3 protein as an apoptotic marker in intact spinal cord, we compared the cC3^+^ cells and cPARP^+^ cells in various experimental contexts. Prior to the analyses, we tested several commercially available antibodies designed against the common apoptotic markers, namely AIF, EndoG, fractin, cC3, cC7, and cPARP. In the analysis, the optimal apoptotic marker(s) should meet two important criteria: specificity for apoptotic cells and discrete signal restricted optimally to the nucleus. Compared to other markers, cPARP proved to be a reliable marker of apoptotic cells, probably owing to its ability to bind newly generated epitopes produced by activated effector caspases during apoptosis (Soldani and Scovassi 2002). Consistent with Soldani and Scovassi (2002), the signal of the p89 fragment of PARP in cPARP^+^ cells can be characterized as perinuclear. As a result of cytoplasmic condensation and cell shrinkage that occur in apoptotic cells, cytoplasmic localization of the p89 fragment of PARP, as shown in Mashimo et al. (2021), cannot be entirely excluded. In contrast to anti-cPARP antibody, the antibodies against the other apoptotic markers did not show the same level of specificity. In primary cell culture, we detected AIF and EndoG in cells with and without morphological changes associated with apoptosis. This can be explained by the fact that during apoptosis, AIF and EndoG only change their localization in the intracellular space and do not generate new epitopes in their structure (Joza et al. 2009; Li et al. 2001). We also evaluated fractin, a cleaved form of actin produced by caspase activity (Rossiter et al. 2000). Although we were able to identify fractin^+^ cells with hallmarks of apoptotic morphology, we found that anti-fractin antibody is probably at least partially immunoreactive to uncleaved actin. Furthermore, we tested an antibody against cC7, another effector caspase involved in apoptosis. We detected cC7 immunoreactivity primarily in the cytoplasm of neurons and neural processes without clear signs of apoptosis. This observation is in line with a previous study that confirmed the general activity of effector caspase-3 and caspase-6 in neural processes (D’amelio et al. 2010). Thus, massive immunoreactivity in the cytoplasm disqualified anti-cC7 antibody for the purpose of detection of apoptotic cells.

According to our quantification of cC3^+^ and cPARP^+^ cells, the spinal cord contains a massive population of cC3^+^ cells with a non-homologous distribution among the studied ROI of spinal cord or among the studied spinal cord ontogenetic phases. The cC3^+^ population was most abundant in the neonatal spinal cord (mainly in white matter) and its number declined significantly in later stages of ontogenesis. The majority of observed cC3^+^ cells did not display apoptotic morphological changes. On the contrary, we identified only a small population of cells containing the p89 fragment of PARP, which is specifically generated during apoptosis (Soldani and Scovassi 2002). In addition, the cPARP population differed from the cC3^+^ cells in size and the distribution of cells across ROI in neonatal spinal cord, with the majority of the cPARP^+^ cells present in the gray matter, especially in the dorsal horns. This localization of cPARP^+^ cells is consistent with the previously identified population of apoptotic neurons (Lawson et al. 1997), which undergo programmed cell death as a result of their inability to be involved in functional neuronal circuits in dorsal horns during the early postnatal period (Prasad et al. 2008). For similar reasons, ablation of superfluous neurons could be expected in other gray matter areas. Intriguingly, we discovered a substantial proportion of cPARP^+^ glial cells showing nuclear alterations in the dorsal funiculus of P90 rats. However, further investigation is required to determine the role of apoptosis in the dorsal funiculus in adulthood. In general, the majority of cPARP^+^ cells displayed hallmarks of apoptosis (semilunar, condensed, or fragmented nucleus), as described by Taatjes et al. (2008).

The accumulation of cC3^+^ cells in the spinal cord (mostly without apoptotic morphology), as well as striking differences in size between cPARP^+^ and cC3^+^ populations, could be explained in two ways: (i) there is a significant time delay in caspase-3 activation followed by PARP cleavage or (ii) cC3 is involved in non-apoptotic processes. On the basis of previous studies, cC3 directly cleaves PARP in the apoptotic pathways (Duriez and Shah 1997; Soldani and Scovassi 2002). Therefore, the presence of cPARP in cells is considered proof of cC3 activity (Li et al. 2000). At the same time, PARP cleavage prevents necrosis during apoptosis, ensuring appropriate execution of caspase-mediated programmed cell death. Therefore, cleavage of PARP by caspases is considered to be a hallmark of apoptosis (Herceg and Wang 1999). PARP cleavage has been observed in early stages of apoptosis, even before DNA degradation in multiple type of tumor cells (Soldani et al. 2001) and cPARP has been referred to as an early apoptotic marker (Kaufmann et al. 1993). However, to our knowledge, the dynamics of cC3 and cPARP in neural cell populations has not been studied before. In order to test our hypothesis, we investigated the time-dependent relationship between the presence of both markers in apoptotic cells. We used primary cell culture prepared from neonatal spinal cord to simulate the heterogeneity of spinal cord containing cell populations at various stages of the cell cycle and/or the level of cell differentiation. In the primary cell culture, we triggered apoptosis by the administration of staurosporine, a well-established apoptotic inductor commonly used in in vitro studies of neuronal apoptosis (Negishi et al. 2003; Prince and Oreland 1997; Yu et al. 2007). Compared with other apoptotic inductors, whose action is based mainly on inducing oxidative stress (Simon et al. 2000) or inhibiting specific enzymes involved in cell proliferation (Park et al. 1997; Wang et al. 2000), staurosporine triggers apoptosis through mechanisms of kinase inhibition (Karaman et al. 2008) and induction of oxidative stress (Kruman et al. 1998). Thus, staurosporine should induce apoptosis in cells regardless of the state of differentiation or cell cycle phase (Bertrand et al. 1994). Owing to the ability of staurosporine to induce caspase-dependent apoptosis (Belmokhtar et al. 2001) resulting in caspase-3 activation and PARP cleavage that could be detected by antibodies, our model proved to be a useful tool for studying the cC3–cPARP time-dependent relationship. On the other hand, higher abundance of Annexin V^+^ cells compared with aC3/7^+^ cells in primary cell culture 7.5 h after staurosporine induction was probably caused by its ability to also induce caspase-independent apoptosis (Belmokhtar et al. 2001), or by an increase in the number of necrotic and necrotic-like cells that are falsely positive for Annexin V in the cell culture (Shlomovitz et al. 2019). Therefore, our model does not represent a universal instrument to study apoptosis in neural cells. Rather, this model provides a specific method for caspase-substrate dynamics research.

In line with data from an in vitro analysis of the impact of staurosporine on the activity of effector caspases-3/7 and the abundance of Annexin V^+^ cells, we found a significant increase in the number of cC3^+^ cells as well as cPARP^+^ cells 4 h after synchronous induction of apoptosis in the primary culture. Therefore, we assume that consecutive activation of caspase-3 followed by PARP cleavage should result in similar sizes of cC3^+^ and cPARP^+^ populations in nervous tissue containing asynchronous populations of apoptotic cells in vivo. Thus, discrepancy in the size of cC3^+^ and cPARP^+^ populations may be caused by the role of cC3^+^ cells in non-apoptotic processes during which PARP cleavage does not occur. Similar observations have been made in neural cells of murine neurospheres, in which cC3 endogenous activity without PARP cleavage was present during differentiation of nestin^+^ precursors to astrocytes, oligodendrocytes, and neurons (Fernando et al. 2005). Increase in the proportion of cC3^+^ oligodendrocytes in our in vitro model even before apoptosis induction and the surprisingly high number of cC3^+^ cells in astroglial and oligodendroglial populations in spinal cord indicate that cC3 may play important non-apoptotic role mainly in glial cells. The presence of cC3 in astrocytes and radial glia like cells has already been reported in multiple studies. Non-apoptotic functions of cC3 have been observed during the process of astrogliosis and astrocyte cytoskeletal remodeling induced by various pathological conditions (Acarin et al. 2007; Aras et al. 2012; Guyenet et al. 2013), or even after non-pathological stimuli like exercise (Stevenson et al. 2018) in various parts of the rodent brain. Caspase-3 activity has also been observed during differentiation of Bergmann glia of the cerebellum in rats (Oomman et al. 2006). In a small population of cC3^+^ neurons, cC3 may participate in several non-pathological processes required for remodeling and establishment of neural networks. Caspase-3 proved to be active during long-term potentiation of synapses in the CA1 region of the rat hippocampus (Gulyaeva et al. 2003), during the long-term phase of synaptic input sensitization in the terrestrial snail *Helix lucorum* (Bravarenko et al. 2006), and in the auditory forebrain of the zebra finch during long-term habituation to tape-recorded birdsongs (Huesmann and Clayton 2006). More intriguingly, it has been proposed that caspase-3 is already present in an active form in zebra finch postsynaptic neurites, and its activity is regulated by the interaction with the apoptotic inhibitor *XIAP* (encoded by the *Birc4* gene) (Huesmann and Clayton 2006). This is consistent with our investigation of IAP gene expression in the rat spinal cord, which revealed that *XIAP* is the most expressed inhibitor of apoptosis in rats at all studied ages. We observed increased transcript levels of *Birc2* that encodes the rIAP-1 protein, whose presence may be explained by fact that the protein is involved in regulation of multiple signal pathways besides the inhibition of apoptosis (Saleem et al. 2013). We also observed elevated levels of *Survivin* (*Birc5*) expression in P8, which is associated with proliferation and should also be capable of binding with cC3 (Shin et al. 2001). Higher expression levels can be explained by higher proliferation rate of cells during the early stages of postnatal development in the spinal cord, which is attenuated in later ages (Alexovič Matiašová et al. 2017). As a result of the ability of both *XIAP* and *Survivin* to bind to cC3 (Riedl et al. 2001), we hypothesize that cC3 is in an activated but still inhibited state. Dynamic dissociation and re-binding of XIAP or Survivin to active site of cC3 probably allow cells to regulate the activity of cC3 spatially and temporally (Huesmann and Clayton 2006). However, additional research is necessary to confirm our hypothesis.

In conclusion, we observed a large population of cC3^+^ cells in the spinal cord during neonatal and preadolescent development and in adulthood that do not correspond to the size of cPARP^+^ cell population, which is considered to be a truly apoptotic cell population. On the basis of data from in vitro analysis of the time-dependent interaction between cC3 and cPARP, we assume that cC3 may be inhibited and/or involved in other non-apoptotic processes. One of the possible explanations is that the activity of cC3 is regulated by the *Birc4 (XIAP)*, *Birc2*, and/or *Birc5 (Survivin)* genes, which potentially could form a “stand-by” complex with cC3. However, further investigation is required to determine the definitive nature of the interaction between IAPs and cC3 in the spinal cord. Our data also demonstrated that cC3^+^ should not be considered as an exclusive apoptotic marker. Other markers/methods should be considered for the detection of apoptotic cells, especially in the intact nervous tissue.

### Supplementary Information

Below is the link to the electronic supplementary material.

Supplementary file1 (DOCX 513 KB)

## Data Availability

The data that support the findings of this study are available from the corresponding author upon reasonable request.
