# Supplementary material for: Cleaved caspase-3 is present in the majority of glial cells in the intact rat spinal cord during postnatal life
Source: Histochem Cell Biol. 2023 Nov 8;161(3):269–86. doi: 10.1007/s00418-023-02249-7 (PMC10912154; doi:10.1007/s00418-023-02249-7)
Supplement: Supplementary file 1 — Supplementary file1 (DOCX 513 KB) [file 418_2023_2249_MOESM1_ESM.docx]

**Cleaved caspase-3 is present in the majority of glial cells in the intact rat spinal cord during postnatal life**

Holota R.^1^, Dečmanová V.^1^, Alexovič Matiašová A.^1*^, Košuth J.^1^, Slovinská L.^2,3^, Pačut L.^1^, Tomori Z.^4^, Daxnerová Z.^1^, Ševc J.^1^

*1- Institute of Biology and Ecology, Faculty of Science, P. J. Šafárik University in Košice, Šrobárova 2, 04154 Košice, Slovak Republic*

*2- Associated Tissue Bank, Faculty of Medicine,P. J. Šafárik University in Košice and L. Pasteur University Hospital, Tr. SNP 1, 04011 Košice, Slovak Republic*

*3- Department of Regenerative Medicine and Cell Therapy, Institute of Neurobiology, Biomedical Research Center, Slovak Academy of Sciences, Šoltésovej 4, 04001 Košice, Slovak Republic*

*4 - Institute of Experimental Physics, Slovak Academy of Sciences, Watsonova 47, 04001 Košice, Slovak Republic*

** Correspondence: anna.alexovic.matiasova@upjs.sk; +421 55 234 1184 Institute of Biology and Ecology, Faculty of Science, P. J. Šafárik University in Košice, Šrobárova 2, 04154 Košice, Slovak Republic*

**Supplementary material**

**
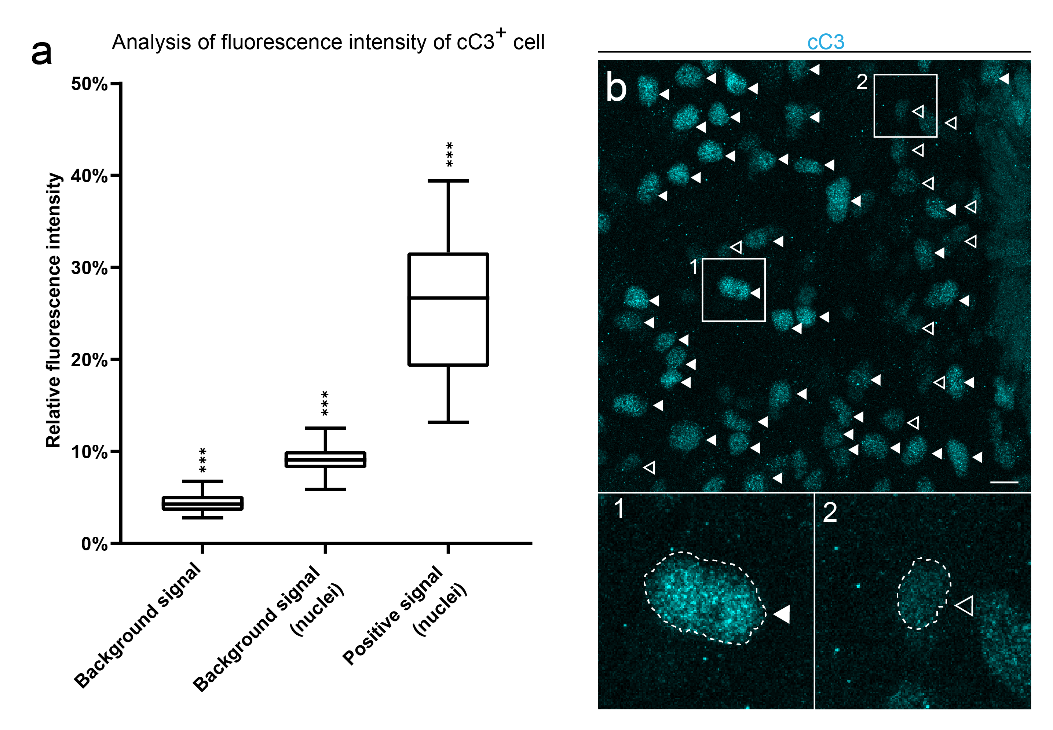
**

**Supplemental Fig. 1 Fluorescence intensity analysis of cC3^+^ nuclei applied to evaluate of cC3 signal in the spinal cord cells and detection of apoptotic cells using the TUNEL assay and cPARP immunolabeling.** To filter potential nonspecific anti-cleaved caspase-3 antibody reported by manufacturer, signal in the spinal cord of rats, relative fluorescence intensity was evaluated on 50 regions without apparent signal (*i.e.*, background signal), 50 nuclei with apparently low intensity signal (*i.e.*, background signal of cell nuclei), and 50 nuclei with apparently high intensity signal (*i.e.*, positive cells). The measured values are expressed as a percentage of brightness density (0% = brightness value 0, 100% = brightness value 255) (a). Based on the results of densitometry analysis, in all microscopy analyses we considered only nuclei with relative fluorescence intensity ≥ 19.4% to be cC3^+^ nuclei. Anything below this intensity was considered either background nuclear or background intercellular signal. The statistical comparison confirmed differences between cells considered positive and nuclear and intracellular background signal (one-way ANOVA). Representative microphotograph of a Z-stack of spinal cord (b) shows highlighted cC3^+^ nuclei (full arrowhead) with a detailed microphotograph of selected positive nuclei (1, arrowhead) and background nuclear signal (2, empty arrowhead).

**Supplemental Table 1 Objectives used in the study**

| IncuCyte^TM^ ZOOM | Nikon Imaging Japan Inc, Tokyo, Japan | PLAN FLUOR 10.0x0.03 DRY |
| --- | --- | --- |
|  |  | ELWD PLAN FLUOR 20.0x0.45 DRY |
| Leica TCS SP5X confocal system | Leica Microsystems, Mannheim, Germany | HC PLAN APO 10.0x0.40 DRY |
|  |  | HCX APO U-V-I 40.0x0.75 DRY UV |
|  |  | HCX PL APO CS 100.0x1.40 OIL |
| Leica Thunder Imager DMi8 microscope |  | HC PL APO 40x 0.95 CORA DRY |

**Supplemental Table 2 Parameters of identification of positive cells using the IncuCyte^TM^ ZOOM system**. During analysis using IncuCyte ZOOM 2016B, the fluorescence intensity of cC3^+^ cells along with signal for cPARP^+^ cells and phenotypic markers was determined with uniform analysis routines prepared by build-in analysis job masks. Because samples had to be submitted to antigen retrieval prior to staining with the anti-cPARP antibody, the routine for phenotype identification was modified specifically due to possible signal changes caused by citrate buffer exposition.

| **Marker** | **Channel** | **Threshold Parameters** | |
| --- | --- | --- | --- |
| Thresholding parameters for live cell analysis | | | |
| Incucyte® Caspase-3/7 Dye for Apoptosis | Green | **Thresholding** | Adaptive, GCU threshold: 0,9000 |
|  |  | **Edge split and sensitivity** | ON, -18 |
|  |  | **Filters** | Maximal area: 1000 µm^2^ |
| Incucyte® Annexin V Dye for Apoptosis | Red | **Thresholding** | Top-Hat, Radius 100µm, GCU threshold: 1,000 |
|  |  | **Edge split and sensitivity** | ON, -30 |
|  |  | **Filters** | No additional filters |
| Thresholding parameters for fixed cell analysis without antigen retrieval | | | |
| Anti-cleaved caspase-3 staining | Green | **Thresholding** | Top-Hat, Radius 100µm, GCU threshold: 0,5000 |
|  |  | **Edge split and sensitivity** | ON, -18 |
|  |  | **Filters** | Maximal area: 1000 µm^2^ |
| Anti-CC-1 staining | Red | **Thresholding** | Top-Hat, Radius 100µm, GCU threshold: 0,5000 |
|  |  | **Edge split and sensitivity** | ON, -28 |
|  |  | **Filters** | Minimal area: 15 µm^2^ Maximal area: 1000 µm^2^ |
| Anti-TUJ1 staining | Red | **Thresholding** | Top-Hat, Radius 100µm, GCU threshold: 0,4000 |
|  |  | **Edge split and sensitivity** | ON, -58 |
|  |  | **Filters** | Minimal area: 20 µm^2^ |
| Thresholding parameters for fixed cell analysis with antigen retrieval | | | |
| Anti-cPARP staining | Green | **Thresholding** | Top-Hat Radius 100µm, GCU threshold: 0,34000 |
|  |  | **Edge split and sensitivity** | ON, -18 |
|  |  | **Filters** | Minimal area: 35 µm^2^ , Minimal area: 1500 µm^2^ |
| Anti-CC-1 staining | Red | **Thresholding** | Adaptive, GCU threshold: 0,1800 |
|  |  | **Edge split and sensitivity** | ON, -28 |
|  |  | **Filters** | Minimal area: 15 µm^2^ Maximal area: 1000 µm^2^ |
| Anti-TUJ1 staining | Red | **Thresholding** | Top-Hat, Radius 100µm, GCU threshold: 0,4000 |
|  |  | **Edge split and sensitivity** | ON, -58 |
|  |  | **Filters** | Minimal area: 20 µm^2^ |

**Supplemental Table 3 Nucleotide sequences of primers used for dPCR**

| Gene (alias)  (GenBank Ref.seq.) | Primer Sequence | Product length |
| --- | --- | --- |
| ***Birc1* (*NAIP6*)**  (XM_008760697) | GGAAACATCAGAAAAGTTTGCCCA | 175 bp |
|  | CACACTGTCATCATCCAATGTCTC |  |
| ***Birc2* (*rIAP1*)**  (NM_021752) | AAAATGCTGACCCTCCAGTTGT | 144 bp |
|  | GGATCTGTCGCTGAACCGTC |  |
| ***Birc3* (IAP1)**  (NM_023987) | TGGCAAAGCAATGGGGTATCA | 110 bp |
|  | CATGGTGAGACGACAGGGAG |  |
| ***Birc4* (*XIAP*)**  (NM_022231) | GGAAGCCAAGTGAAGACCCTT | 143 bp |
|  | TTCAGCAGTTCTTACCACAGAT |  |
| ***Birc5*** (*Survivin*)  (NM_022274) | CATCCACTGCCCTACCGAGA | 156 bp |
|  | TCTTCCACCTGCTTCTTGACT |  |
| ***Birc6***  (NM_001170596) | AAACCACTGGGGGTCATAGC | 151 bp |
|  | TGGACAGAAACCAACACTTGC |  |
| ***Birc7***  (NM_001305210) | AGGATCTCCGCTTAGCCTCC | 142 bp |
|  | CAGCTCTGTAGACCCCCGTA |  |
| ***Eef1a1***  (NM_175838) | TGCTGGAGCCAAGTGCTAAT | 181 bp |
|  | GTGCCAATGCCGCCAATTTT |  |
| ***Gapdh***  (NM_017008) (Bangaru et al. 2012) | AGACAGCCGCATCTTCTTGT | 142 bp |
